# Application of Neural Networks to Analyse the Spatial Distribution of Bicycle Traffic Before, During and After the Closure of the Mill Road Bridge in Cambridgeshire, United Kingdom

**DOI:** 10.3390/s25103225

**Published:** 2025-05-20

**Authors:** Shohel Amin

**Affiliations:** Department of Engineering Management, Aston University, Birmingham B4 7ET, UK; s.amin15@aston.ac.uk; Tel.: +44-121-204-4685

**Keywords:** traffic sensors, traffic data, spatial analysis, ANN, bicycle, bridge closure

## Abstract

Traffic congestions due to construction and maintenance works of road infrastructure cause travel delays, unpredictability and less tolerant road users. Bicyclists are more flexible with road closures, shifting to alternative routes, public transport and other active transport depending on the infrastructure, quality and transport services. However, the mixed traffic environment near road closures increases the safety risks for bicyclists. Traditional traffic monitoring systems rely on costly and demanding intrusive sensors. The application of wireless sensors and machine learning algorithms can enhance the analysis and prediction ability of traffic distribution and characteristics in the proximity of road closures. This paper applies artificial neural networks (ANNs) coupled with a Generalised Delta Rule (GDR) algorithm to analyse the sensor traffic data before, during and after the closure of the Mill Road Bridge in Cambridge City in the United Kingdom. The ANN models show that the traffic volume of motorbikes (44%) and buses (34%) and the proximity of Mill Road Bridge (39%) are significant factors affecting bicycle traffic before the closure. During the bridge closure, the proximity of the bridge (99%) and traffic volume of large rigid vehicles (51%) are the most important factors of bicycle distribution in nearby streets leading cyclists to unsafe detours. After the reopening of the Mill Road Bridge, unclear signage caused continued traffic impact, with motorbikes (17%) and large vehicles (24%) playing the most significant role in the spatial distribution of bicycle traffic. This paper emphasises safety concerns from mixed traffic and highlights the importance of cost-effective sensor-based traffic monitoring and analysis of the sensor data using neural networks.

## 1. Introduction

The accurate and timely detection of traffic congestion is important for properly informing road users and employing effective mitigation strategies. Identification of congestion clusters is a cost-effective and efficient approach to monitor and forecast traffic flow. Instead of deploying expensive data collection systems across an entire road network—including areas that rarely experience congestion during the closure of a particular road or transport infrastructure—sensors prioritise key locations where traffic bottlenecks frequently occur. By identifying and analysing these congestion-prone areas, traffic management systems can understand the traffic hotspots of road networks. Effective monitoring and predictive modelling of congestion hotspots can provide the following: (1) adaptive traffic control measures, such as dynamic lane management, optimised signal timing and congestion pricing; (2) more relevant and timely updates about congestion hotspots, enabling better route planning and reducing unnecessary delays; and (3) network-wide insights to infer and predict conditions across the larger network [1].

To develop an effective mitigation measure, it is important to understand recurrent congestion (RC) and nonrecurrent congestion (NRC) [2,3,4]. The RC event is the predictable and routine congestion that occurs at the same places and times every day due to high traffic volume exceeding road capacity, particularly during rush hours. Nonrecurrent congestion occurs due to unexpected events that disrupt normal traffic patterns such as traffic accidents, construction and maintenance works, weather conditions and special events. These disruptions are temporary but can cause severe delays. The occurrence of an NRC event creates frustration not only for individual travellers but also for businesses and organisations because it is a significant source of variability in travel times and is unpredictable in nature [5]. Travellers are less tolerant of these delays due to serious consequences such as being late for work, missing important meetings or incurring unexpected expenses like childcare fees [6]. The negative emotional and financial impacts of these delays are felt more acutely in urban environments where traffic congestion is already a significant concern.

Cyclists and other active commuters are more flexible in responding to NRC events. The spatial distribution of bicycle traffic during road closures is influenced by the availability of alternative routes, cyclists’ adaptability and the quality of cycling infrastructure. Cities that proactively plan for bicycle traffic during closures by implementing temporary infrastructure and wayfinding measures can mitigate disruptions [7]. Hood et al. [7] found that cyclists tend to shift to parallel low-traffic streets or dedicated bike lanes when primary roads are closed. Similarly, Wang and Nihan [8] developed a simulation model demonstrating that bicycle traffic tends to redistribute towards pre-existing cycling infrastructure rather than general-purpose detours. While many cyclists adapt by finding alternative routes, some opt for different modes of transportation. Piatkowski et al. [9] found that, during prolonged road closures, a segment of the cycling population shifts to public transit, particularly when transit options offer competitive travel times. However, Buehler and Pucher [10] argued that, in cities with robust cycling infrastructure, road closures often result in temporary increases in bicycle mode share, as some individuals switch from cars to bicycles due to congestion. Aldred and Goodman [11] stated that prioritisation of non-motorised traffic could lead to an increase in cycling volumes due to improved safety and comfort. However, unplanned closures, such as those caused by construction, often lead to a decline in cycling without adequate cycle-friendly detour routes [12].

The existence of motorised vehicles and heavy goods vehicles (HGVs) poses significant risks to cyclists due to their size, blind spots and manoeuvrability challenges in the proximity caused by road closures. Mindell et al. [13] observed that the presence of HGVs on urban roads correlated with an increase in stress and accident risk, leading some cyclists to avoid certain routes. Furthermore, research suggests that areas with high HGV traffic experience a lower rate of cycling, as cyclists perceive these zones as unsafe [14]. The redistribution of bicycle traffic following road closures or increased HGV presence depends on several factors, including the mixed-traffic environment, infrastructure design, alternative route availability and cyclist perception of safety [15].

Because NRC events are unpredictable, cities need agile and responsive strategies to minimise their impacts, such as (1) real-time traffic monitoring using sensors, cameras and Global Positioning System (GPS) data to detect disruptions and alert road users; (2) incident management systems deploying quick-response teams; (3) dynamic traffic routing using navigation apps helping road users to avoid congestion by suggesting alternative routes; (4) adaptive traffic signals that adjust in real time based on traffic conditions; and (5) better roadwork planning by scheduling maintenance during off-peak hours and providing advanced notice to drivers. However, extensive and timely data collection is required to understand the effects of NRC events on travel time, congestion and the surrounding road network [3]. The application of GPS-enabled devices and sensor networks has enabled continuous and comprehensive traffic monitoring by providing data for traffic flow modelling, congestion prediction and improved traffic management strategies. Machine learning and artificial intelligence further enhance traffic analysis by identifying patterns, forecasting congestion and optimising traffic signal control.

Traditional traffic surveillance systems rely on intrusive sensors embedded or placed in the road surface to monitor traffic flow. These intrusive sensors are effective in capturing various traffic parameters, such as vehicle count, speed and vehicle classification [16]. Since these systems depend heavily on road structure, successful deployment requires detailed knowledge of the transportation network’s layout, traffic volume projections and future urban development. However, urbanisation and the changing pattern of traffic limit the adaptability and effectiveness of these systems. These sensors are subjected to constant wear and tear from traffic, weather conditions and other environmental factors. To replace these sensors, roads require digging up, causing significant traffic disruptions. This causes delays and economic losses due to time and costs associated with road closures and construction work. Flexible, adaptive and nonintrusive technologies, such as wireless sensors, provide significant benefits in urban environments, where road networks are evolving and congestion is unpredictable.

However, the variability and big data size of sensor traffic data make it challenging to develop a single model capable of accurately analysing NRC traffic distribution across the selected roads. Efficient methods are essential for analysing distribution patterns of motorised and non-motorised traffic in road networks in the proximity of NRC events. The ANN predicts the results with greater accuracy and determines the importance of explanatory variables, reducing statistical errors [17,18]. Application of ANN requires built-in functions and training parameters, such as learning rate and momentum term [19]. ANNs are very effective for analysing nonlinear traffic data for their ability to model complex relationships of high-dimensional inputs and adapt to evolving traffic patterns. ANNs can generalise historical data to predict future traffic flow, speed and congestion by adapting to different road environments and data from sensors, GPS and cameras. ANNs use multiple hidden layers with many neurons to learn complex patterns across dimensions, where each neuron learns a different combination or interaction of features. These hidden layers act as filters for transforming high-dimensional inputs into meaningful and compact internal representations. This paper uses the sensor traffic data to analyse the spatial distribution of bicycle traffic before, during and after the closure of the Mile Road Bridge in Cambridge City in the United Kingdom by integrating ANN and GDR algorithms. The GDR algorithm utilises a constant negative gradient of the error paraboloid surface to determine the stability and speed of convergence of weight vector, minimising the error value [19]. It updates the weights during training by allowing ANN to adjust weights not only for the output layer but also for the hidden layers. The GDR applies the principle of gradient descent optimisation to compute the gradient of the error function and propagates it backwards to converge towards a local minimum of error function and adjust the weights in all layers to achieve low error rates. Integration of GDR with ANN is very effective as networks grow in depth and complexity, allowing for scalable solutions in deep learning.

This paper is organised as follows. Section 2 discusses the relevant literature on traffic sensors and spatiotemporal clustering of motorised and non-motorised traffic in NRC events. Section 3 analyses the sensor traffic data using ANN models to understand the spatial distribution of bicycle traffic before, during and after the closure of Mill Road Bridge. Section 4 describes the results of ANN models for the spatial distribution of bicycle traffic in three scenarios. Section 5 discusses the importance of variables for spatial distribution of bicycle traffic in three scenarios. Finally, Section 6 concludes this paper by summarising the modelling outputs and stating the limitations and future research directions.

## 2. Literature Review

The advancements in Micro-Electro-Mechanical Systems (MEMS) revolutionised the field of sensor technology, opening new possibilities for improved traffic monitoring, congestion detection and road network management [20,21,22,23]. By integrating the sensing, processing and communication capabilities into a single device, MEMS sensors provide a more efficient, cost-effective and scalable method for monitoring urban traffic. The real-time data processing, enhanced accuracy and ability to detect NRC events improve traffic flow management in NRC events. Zheng et al. [20] used the Honeywell HMC5883L magnetic sensor (manufactured by the Honeywell International Inc. in the United States of America) and the Zigbee wireless protocol in the wireless sensor network to filter the data and apply the decision-making algorithms in calculating traffic flow. However, the raw data collected by Honeywell HMC5883L magnetic sensors are often subjected to noise, environmental interference and anomalies. Zheng et al. [20] used the Zigbee filtering algorithms to clean and refine the data, ensuring that only relevant and accurate vehicle information is retained for further analysis. After filtering data, Zheng et al. [20] applied decision-making algorithms to process data and make decisions on traffic flow. However, the reliability of Zigbee network must be maintained to ensure that data are transmitted accurately and in real time.

The University of California, Berkeley, used magnetic and acoustic sensors to accurately identify vehicles in various environmental conditions [24,25]. After evaluating both sensing methods, magnetic sensors were identified as the preferred choice. They were installed at the centre of highways or intersections, with an installation time of approximately 10 minutes [24,25]. The system had a strong accuracy rate (around 80%) but needed to interrupt traffic, causing delays, safety concerns and operational challenges.

Several studies utilised data collected by wireless sensor networks (WSNs) to classify vehicles [26]. The WSNs, made up of a distributed network of small and low-power sensors, can be deployed along roads or highways to collect real-time data, such as vehicle counts, speeds and types. The sensors are typically equipped with technologies like radar, infrared and ultrasonic sensors to detect the presence and movement of vehicles. Types of vehicles can be identified based on various characteristics such as size, weight, speed and axle configuration.

Machine learning algorithms and advanced statistical models are frequently utilised to process real-time data streams to identify patterns, detect anomalies and generate short- and long-term traffic predictions. De Fabritiis et al. [27] and Herring et al. [28] demonstrated the potential of leveraging mobile sensor data for real-time traffic estimation and predictive analytics. The traffic forecasting methodologies were improved with spatiotemporal models; those captured both spatial dependencies (how traffic affects nearby roads) and temporal dynamics (how traffic changes over time). Nguyen et al. [29] investigated the propagation of traffic congestion across road networks over time by constructing causality trees (CCTs) from congestion data in Sydney central business district (Australia). Min and Wynter [30] developed a multivariate spatial–temporal autoregressive model to understand the traffic interactions between various types of roads. Kamarianakis and Prastacos [31] used the space–time autoregressive integrated moving average (STARIMA) model to analyse spatial and temporal traffic flow and demonstrated the importance of considering temporal and spatial correlations. Yue and Yeh [32] studied the spatiotemporal characteristics of highway traffic flow, providing insights into congestion formation and propagation. Cheng et al. [33] investigated correlations between links in the London traffic network, highlighting the complexity of STARIMA in effectively capturing traffic dependencies. By understanding the propagation of traffic congestion across a network, traffic managers can make more precise predictions and implement targeted interventions.

Road closures, whether planned or unplanned, significantly impact the spatial distribution of traffic. Understanding these impacts is crucial for urban planning, traffic management and emergency response. Studies indicate that traffic redistribution during road closures follows predictable patterns based on road network connectivity, availability of alternate routes and traffic characteristics. Zhang et al. [34] stated that traffic diversities across secondary and tertiary roads often resulted in congestion in unexpected areas when a major arterial road is closed. Similarly, Papageorgiou et al. [35] found that detour routes absorbed varying traffic loads depending on their capacity and the presence of real-time traffic information systems.

Several factors influence traffic redistribution during a closure, such as topology and availability of alternative routes [36], signal timing adjustments and dynamic traffic management in rerouted areas [37], road users’ familiarity with alternative paths, real-time information and perceived travel time [38]. Several case studies highlighted the significance of understanding traffic redistribution. For example, a study on the I-405 freeway closure in Los Angeles demonstrated that proactive traffic management strategies reduced congestion by 20% [39]. In contrast, an unplanned road closure in London led to severe disruptions due to inadequate detour planning [40]. Studies employing GIS-based models [41] showed that cyclists seek alternative routes with dedicated cycling infrastructure after encountering disruptions despite increase of distance. The emergence of low-traffic neighbourhoods (LTNs) has further demonstrated that strategic road closures could redistribute cycling demand towards safer and less congested streets [42]. The interaction between road closures, HGV traffic and bicycle route choice is complex but has significant implications on urban mobility. This paper applies ANN models to analyse the impact of mixed traffic environment, distance from the Mill Road Bridge and traffic characteristics on the spatial distribution of bicycle traffic before, during and after the closure of the Mill Road Bridge.

## 3. Materials and Methods

### 3.1. Data Collection and Case Study

**Figure 1:**
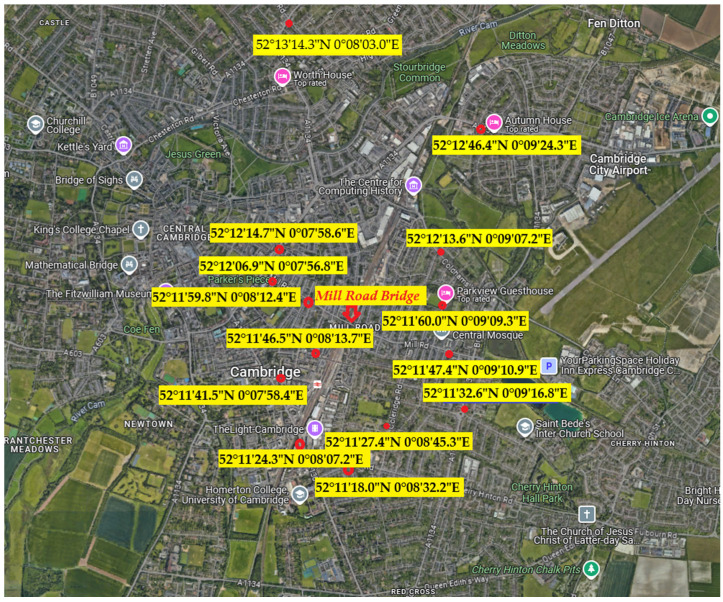
Locations of the Mill Road Bridge and sensors.

The Cambridge City Council conducted the Mill Road Sensor Project to monitor the impact of an eight-week closure of the Mill Road Bridge in 2019 during crucial rail service improvements by Govia Thameslink railway operating Southern, Thameslink, Great Northern and Gatwick Express. To better understand traffic patterns and environmental conditions, the Smart team installed 15 VivaCity traffic sensors (Figure 1 and Table 1) and the City Council deployed seven air quality sensors on and around Mill Road Bridge. These sensors are created by VivaCity Labs Limited in London, United Kingdom and were set up to monitor changes in road usage and air quality, particularly in response to the closure of Mill Road Bridge and its impact on surrounding streets. The sensors were originally installed in May 2019 for 18 months to gather data on pedestrians, bicycles, cars and other vehicles. This allowed the council to assess the closure’s impact on surrounding roads and air quality. Pedestrians and cyclists could cross via a temporary footbridge, except for a brief five-day period when the route was closed for safety. Although the initial monitoring period was set for 18 months, data collection via these sensors is still ongoing and is now part of a broader city-wide monitoring network aimed at improving urban planning, traffic management and environmental policies. The sensors collected hourly traffic data on the selected roads from 3rd June 2019 midnight to 13th December 2020 midnight (Table 1).

### 3.2. ANN Models for Bicycle Distribution on Roads in Proximity to the Mill Road Bridge

Integration of big data and neural networks provides the vast and varied information necessary to train robust neural models, while neural networks offer sophisticated tools to uncover hidden patterns and effectively analyse big data. As both fields continue to evolve, their integration drives further innovations across transport disciplines. Neural networks can automatically learn and extract features from raw data that are especially valuable when dealing with the high dimensionality of big data. Application of ANN in analysing big data can improve the accuracy of forecasting models by identifying anomalies and making decisions based on complex data patterns. The ANN with GDR algorithm manages nonlinear, multidimensional relationships, focusing on postprocessing uncertainty for precise model parameters. This paper couples the ANN and GDR learning algorithm to analyse the spatial distribution of bicycle traffic using the sensor data. Traditional ANN models might face challenges with the complex and nonlinear nature of traffic data. The GDR learning algorithm addresses these challenges by acknowledging the multi-dimensional and unpredictable characteristics of traffic data, thereby reducing ambiguity and enhancing analytical accuracy. This paper builds an ANN model (Equation (1)) using the derivative of sigmoid activation function ($\overset{´}{\sigma }\left(l\right)$) to effectively model positive values and improve computational efficiency during the training process of bicycle traffic data (Equations (2) and (3)).

(1) $$z^{l}=W^{l}a^{l-1}+b^{l},\;a^{l}=\sigma (z^{l})$$

(2) $$\overset{´}{\sigma }\left(l\right)=\sigma (l)\cdot (1-\sigma \left(l\right))$$

(3) $$\sigma \left(l\right)=\frac{1}{1+e^{-l}}$$

where $z^{l}$ is the input to the activation function (can be scalar or vector) at layer *l*, $W^{l}$ is the weight matrix for layer *l*, $\sigma (z^{l})$ is the output (activation) at layer *l*, $a^{l}$ is the activation for layer *l*, $a^{l-1}$ is the activation from the previous layer, $b^{l}$ is the bias vector for layer *l*, σ(l) is the sigmoid activation function and $\overset{´}{\sigma }\left(l\right)$ is the derivative of the sigmoid activation function.

**Figure 2:**
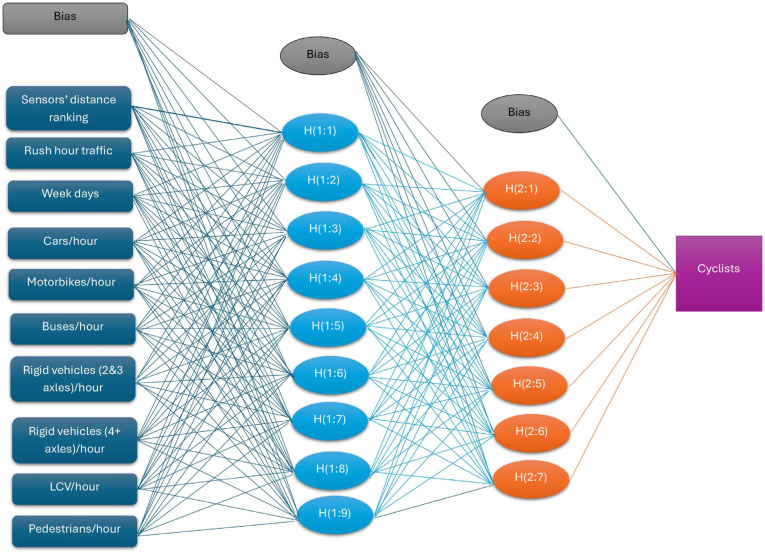
ANN diagram for spatial distribution of bicycle traffic.

The synaptic weights ($W^{l})$ of ANN models represent the strength of connections between neurons in different layers (Equation (4)). These weights determine how signals (inputs) are transferred from one layer to another and influence the network’s ability to approximate functions or solve specific tasks. The synaptic weight can be positive or negative and it determines whether the output of a neuron is strengthened (positive weight) or diminished (negative weight) as the input signal passes through. Positive weights increase the likelihood that a neuron will “fire” or become activated, potentially contributing to the output in a favourable direction. On the other hand, negative weights suppress the activation of neurons and reduce the output, thus helping the network learn to refine its predictions by reducing undesired responses.

(4) $$W^{l}≔W^{l}-\alpha \cdot \delta^{l}\cdot {\left(a^{l-1}\right)}^{T}$$

where α is the learning rate, $\delta^{l}$ is the error signal at layer *l* and ${\left(a^{l-1}\right)}^{T}$ is the transpose of $a^{l-1}$. The errors of ANN models are calculated by comparing the predicted output with the desired output (often through a loss function, such as Mean Squared Error) (Equation (5)). The error is then propagated back through the network, starting from the output layer to the hidden layers, adjusting weights using the gradient descent method (Equation (6)). Each layer’s contribution to the error is estimated and the weights are updated accordingly. This cycle is repeated iteratively across training examples, with the weights being adjusted each time. With each iteration, the model becomes better at approximating the desired outputs and learning the underlying patterns in the data. This is the general process that enables neural networks to learn from data and improve their accuracy over time.

(5) $$\delta^{l}=(a^{l}-y)⨀\overset{´}{\sigma }(z^{l})$$

(6) $$\delta^{l}={\left(W^{l+1}\right)}^{T}\delta^{l+1}⨀\overset{´}{\sigma }(z^{l})$$

where ⨀ represents element-wise multiplication and *y* is the observed output. Figure 2 shows the input, hidden and output layers of ANN models for bicycle traffic. The distance of sensors from the road closure, presence of rush hour traffic, days of the week (weekdays or weekends/bank holidays) and mixed traffic characteristics (number of cars, buses, pedestrians, other good vehicles and light commercial vehicles) are the neurons in the input layer. The first and second hidden layers have nine and seven hidden neurons (synapse layers) represented by the synaptic weights yielding towards the decision boundary, respectively (Figure 2). The magnitude of a synaptic weight is equivalent to a combination of increased resembling connections between different components of layers (such as input variables and synapse layers of hidden layers). The positive weights increase the prospect of receiving a cell with an action potential, while negative weights have the opposite action (Figure 2). The outputs are predicted to apply a two-phase propagate–adapt cycle and compared with the desired outputs to estimate the errors (bias). These errors are transferred backward from the output layer to synapse layers in hidden layers roughly based on the relative contribution of synapse layers to the estimated output. This process was repeated layer by layer until each neuron in the network received an error representing its relative contribution to total errors.

## 4. Results

The dataset collected by the VivaCity sensors before, during and after the closure of Mill Road Bridge was systematically divided into three subsets, such as training, testing and validation data, in order to develop and evaluate the ANN models for the spatial distribution of bicycle traffic. The primary objective of this partitioning is to ensure that ANN is effectively trained to avoid overfitting. The training datasets constituted approximately 48% of the total data for three scenarios. The training datasets were used to adjust the models’ internal parameters, such as weights and biases, through iterative learning (Table 2). The network learns patterns and relationships within the data to optimise its predictive capability. The testing datasets comprised of about 29% to 32% of the total data for three scenarios played a crucial role in monitoring the network’s performance during the training processes (Table 2). By tracking errors in the testing data, the model can prevent overfitting, ensuring that it generalises well to unseen data rather than memorising the training data. Finally, the validation datasets, accounting for the remaining 20% to 24% of the total data for three scenarios, were utilised to assess the overall predictive performance of the trained ANN models (Table 2). This final evaluation step ensures that the network’s learned patterns are reliable and applicable to new inputs. This systematic data partitioning approach helped in building accurate and generalisable ANN models capable of effectively predicting the spatial distribution of bicycle traffic based on the input features.

### 4.1. Performance Evaluation of ANN Models

This study evaluates the performance of ANN models to determine their statistical significance. The fitness and predictive accuracy of ANN models were assessed using two key error metrics, such as sum of squares error (SSE) and relative error (RE), respectively (Table 3). The SSE represents the total discrepancy between the predicted and actual values. When the sigmoid activation function was applied to the output layer, the SSE corresponded to the cross-entropy error, a widely used loss function in classification problems. During training, ANN models aimed to minimise SSE or improve the network’s ability to make accurate predictions.

The RE quantifies the percentage of incorrect predictions and is directly related to the dependent variable. RE is calculated as the ratio of SSE for the dependent variable and SSE of a “null model”. A lower RE value indicates better model performance, suggesting that ANN models provide more accurate predictions compared to a model with no predictive capability. By analysing these criteria, this study ensures that ANN models for three scenarios are both statistically significant and effective in making reliable predictions.

The estimation of ANN models shows an insignificant difference between the predicted values obtained from the estimators and the actual output values (Table 3). This indicates that the models had effectively learned the underlying patterns in the training data with minimal estimation errors. Similarly, the testing datasets used to track errors during the training process and to prevent overfitting exhibited a low expected value of squared error loss (Table 3). This suggests that models maintain good generalisation capability without excessive deviation from the true values. Furthermore, the validation datasets that serve as a final assessment of models’ predictive performance also demonstrated insignificant errors (Table 3). This further confirms the high accuracy and reliability of the trained ANN models in making precise predictions.

**Figure 3:**
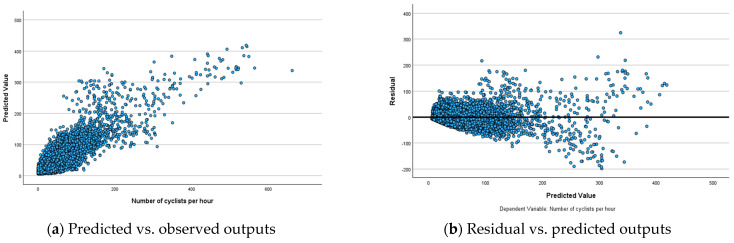
Relationships among observed, predicted and residual outputs for ANN model before the closure of the Mill Road Bridge.

**Figure 4:**
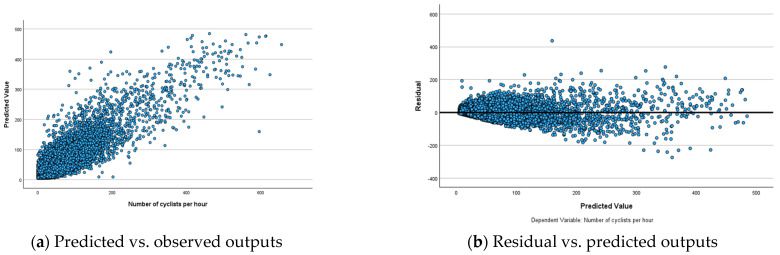
Relationships among observed, predicted and residual outputs for ANN model during the closure of the Mill Road Bridge.

**Figure 5:**
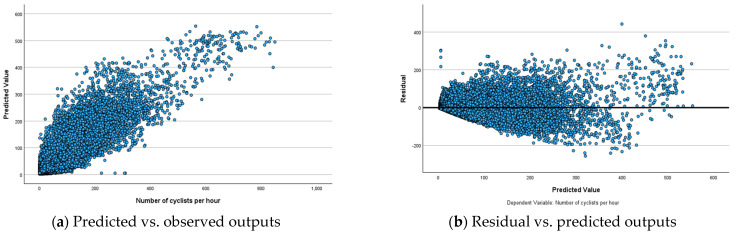
Relationships among observed, predicted and residual outputs for ANN model after the closure of the Mill Road Bridge.

To analyse the performance of ANN models, predicted-by-observed and residual-by-observed scatterplots were generated (Figure 3, Figure 4 and Figure 5). These visualisations help in understanding the relationships between predicted and observed values and residuals and observed values, respectively. In the predicted-by-observed scatterplots, the y-axis represents the predicted number of bicyclists, while the x-axis represents the observed number of bicyclists for the combined training and testing datasets before (Figure 3a), during (Figure 4a) and after (Figure 5a) scenarios of the Mill Road Bridge closure. Ideally, the data points should align closely along a 45-degree line originating from the origin, indicating that the model’s predictions are accurate and unbiased. The scatterplots for before, during and after scenarios reveal that ANN models perform reasonably well in predicting the amount of bicycle traffic (Figure 3a, Figure 4a and Figure 5a). The clustering of points along the 45-degree reference line suggests that ANN models successfully captured the underlying relationships within the datasets demonstrating strong predictive accuracy.

The residual-by-observed scatterplots were generated to analyse the distribution of residuals in relation to the predicted bicycle traffic (Figure 3b, Figure 4b and Figure 5b). In these plots, the *y*-axis represents the residuals, while the *x*-axis represents the predicted values of bicycle traffic. This visualisation helps assess the overall fit and behaviour of the network’s predictions. The residual-by-predicted scatterplots for three scenarios exhibit a well-behaved and randomly scattered pattern, indicating a good model fit. ANN models effectively captured the underlying relationships for these scenarios without significant bias. The residuals formed a horizontal band and randomly fluctuated around the zero-line, indicating well-fitted models with few outliers (Figure 3b, Figure 4b and Figure 5b). However, these outliers did not significantly affect the estimation of ANN models, ensuring the robustness of overall model performance.

### 4.2. Parameter Estimation of Input Variables

The predictive variables (input features) were initially fed into the input layer of ANN models. These inputs propagate to the hidden layers, which process the data and help extract complex patterns. This paper employed the Multi-Layer Perceptron (MLP) network to predict an output value by using a set of input variables. The MLP network is essentially a feedforward neural network to find out the optimal relationship between inputs and outputs by minimising the prediction errors. To achieve the best predictive accuracy, the MLP minimises the prediction error of its outputs. The MLP network was trained to optimise the weights such that the output predictions are as close as possible to the actual values. This involved adjusting the weights iteratively through an optimisation procedure. The MLP procedure computed the minimum and maximum values of a given range to help determine the appropriate number of hidden layers for minimising errors. Bayesian Information Criterion (BIC) was used to evaluate the model’s fitness and complexity. By minimising both the prediction error and BIC, the MLP aimed to find the most efficient model.

The best-performing configuration was two hidden layers for the ANN models for three scenarios, as two hidden layers achieved the optimal balance between complexity and predictive accuracy (Table A1, Table A2 and Table A3). In the first hidden layer, the input data are distributed into three sub-layers, denoted as H(1:1), H(1:2) and H(1:3) (Table A1, Table A2 and Table A3). These sub-layers represent different processing units that further refine the input data to capture various features or interactions in the datasets. Multiple sub-layers can enhance the network’s ability to model complex relationships. In addition, the sigmoid activation function was applied to the hidden layers. The sigmoid function transformed the input to the hidden layer into an output, and its Gaussian “bump” property helped smooth transition between inputs and output. The Gaussian “bump” refers to the shape of the curve that the sigmoid function created, helping the network capture underlying patterns in the data.

## 5. Discussion

The ANN models conducted sensitivity analyses to assess the importance of input variables in determining the spatial distribution of bicycle traffic based on the combined training and testing datasets (Table 4, Table 5 and Table 6). The importance of an input variable is measured by how much the bicycle traffic changes as the value of a particular input variable changes. This allows the most influential factors for bicycle traffic to be identified. The spatial distribution of bicycle traffic was primarily determined by the hourly volume of motorbikes (44%), buses (34%) and sensors’ proximity to the Mill Road Bridge before the closure of the bridge (Table 4). Interestingly, the spatial distribution of pedestrians is equally affected by the input variables of bicycle traffic for three scenarios (Table 4, Table 5 and Table 6).

During the closure of the Mill Road Bridge, the most important variable was the sensors’ proximity to the Mill Road Bridge (99%), followed by number of large rigid vehicles (four axles and more) per hour (51%) (Table 5). Bicyclists were forced to take alternative routes, resulting in longer travel times and less direct paths. If detour routes are not bike-friendly, such as roads with high commercial and large vehicles, lack of bicycle lanes and unsafe conditions, cyclists might face increasing safety risks and discomfort during their ride. In addition, the narrow and congested alternative routes could create conflict between bicycles and vehicles, potentially leading to accidents and delays.

The sensors’ proximity to the Mill Road Bridge was still contributing to the spatial distribution of bicycle traffic after the opening of the Mill Road Bridge (Table 6). There might be insufficient or unclear signage indicating that the road is now open to traffic or directing cyclists and other road users to appropriate routes. Cyclists might still assume that the road was closed or dangerous, leading them to avoid the area or continue using detour routes even though the road is safe for cycling again. Moreover, the volumes of motorbikes (17%) and large vehicles with two and three axles (24%) significantly affected bicycle traffic after the opening of Mill Road Bridge (Table 6). In general, cyclists face significant safety risks when riding in mixed traffic of motorcycles and large vehicles. These risks include limited visibility, speed discrepancies and unpredictable manoeuvres, especially with trucks and buses. Cyclists may also be affected by air turbulence from large vehicles and unsafe road conditions. To improve safety, dedicated bike lanes, reduced speed limits, cyclist and driver education, clear signage and stricter traffic law enforcement are essential. These measures can help reduce accidents and ensure a safer environment for cyclists in mixed traffic conditions.

## 6. Conclusions

The variability and big data size of sensor traffic data make it challenging to develop a single model capable of accurately analysing the nonrecurrent traffic distribution across the selected roads. Efficient methods are essential for analysing distribution patterns of motorised and non-motorised traffic in road networks near NRC events. The ANN can predict results with greater accuracy and determine the importance of explanatory variables without statistical errors in interpreting the traffic distribution. This paper builds on the demonstrated advantages of using ANN for traffic data analysis and integrates the GDR algorithm to analyse the sensor traffic data before, during and after the closure of the Mill Road Bridge.

The ANN models estimated that motorbike volume (44%), bus volume (34%) and proximity to the bridge were the most important factors influencing bicycle traffic distribution before the Mill Road Bridge closure. During the closure, proximity to the bridge (99%) and large rigid vehicle volume (51%) were the most significant factors forcing cyclists onto less safe detours. After reopening, proximity to the bridge still influenced traffic, possibly due to unclear signage. Motorbike (17%) and large vehicle (24%) volumes also impacted cyclists. This study highlights safety risks from mixed traffic and suggests dedicated bike lanes, speed limits, education, signage and stricter traffic laws to improve cyclist safety. Several studies suggested the cost-effectiveness of dedicated bike lanes across public safety, health, economic development and environmental impact. The New York State Department of Transportation [43] observed that dedicated bike lanes reduced the injuries of all road users by up to 40%. Similarly, Kiani et al. [44] observed that the expansion of cycling infrastructure, including dedicated bike lanes, was associated with increased cycling rates in Montreal, Canada. Macmillan et al. [45] estimated that investing NZD 10 million in cycling infrastructure in New Zealand returned over NZD 24 million in healthcare savings due to increased physical activity that resulted in NZD 2.50 to NZD 5 returns in health and productivity benefits for every NZD 1 investment in bicycle infrastructure in New Zealand [45]. Monsere et al. [46] found that bicycle lanes could improve business with minimal negative impact on sales and employment in five cities of the United States of America, such as Austin (Texas); Chicago (Illinois); Portland (Oregon); San Francisco (California); and Washington (District Columbia).

The findings highlight the importance of accurate traffic congestion detection for traveller information systems and traffic control. This paper focuses on congestion clusters, emphasising a cost-effective approach using sensors to monitor key locations rather than the entire road network. By leveraging neural networks, the research aims to enhance traffic monitoring and forecasting, providing insights into traffic flow changes due to infrastructure modifications. However, several factors affect how traffic redistributes when a road closure occurs. The structure of the road network determines how easily traffic can reroute. A grid-like system offers more alternative paths than a network with limited connections. If multiple roads can absorb diverted traffic, congestion may be less severe. If options are limited, bottlenecks are more likely. Traffic lights can be adjusted to improve flow on detour routes, reducing congestion in affected areas. Tools like variable message signs, smart traffic signals and ramp metering can help regulate traffic flow and minimise delays. Notably, sensors such as inductive loop detectors, radar, cameras and Bluetooth trackers enable data-driven decision-making systems for dynamic traffic redistribution. These systems analyse traffic conditions such as vehicle speed, density and queue length and automatically adjust traffic light timings, update variable message signs and integrate with navigation systems to reroute traffic. The continuous flow of sensor data enables rapid responses to congestion, ensuring minimal delays and improving the safety of both road users and workers. This adaptive approach results in more efficient traffic management and safer work zone environments. However, the loss of data, inaccurate traffic reporting and increased traffic congestion may result from hardware and power failures, signal interference, software and calibration errors and environmental obstructions to traffic sensors. It is important to carefully plan and place sensors and supplement and combine data from vehicles, drones and smartphone devices. The traffic management systems of local councils can use a combination of data filtering, sensor fusion, redundancy and advanced analytical tools to ensure robustness and actionable traffic data. Regular sensor maintenance, along with real-time monitoring, can further reduce the impact of these challenges.

Future studies should focus on including these challenges within ANN models predicting the spatial distribution of bicycle traffic before, during and after the closure of a road or transport sub-system. Cities that plan proactively for bicycle traffic during road closures can minimise disruptions by temporarily adjusting infrastructure to accommodate cyclists. This might include installing bike lanes, rerouting paths and creating temporary bike-sharing stations. Additionally, wayfinding measures, such as clear signage and digital apps, can guide cyclists safely around the closure areas. These efforts help ensure that cycling remains a convenient, safe and sustainable mode of transport even when regular routes are interrupted.

## Figures and Tables

**Table 1:** Location of sensors and proximity ranking from the Mill Road Bridge.

Reference	Sensor Location	Latitude	Longitude	Proximity Ranking
[1]	362 Mill Rd	52.19651	0.15303	11
[2]	Mill Rd (SO 1 Mortimer Rd)	52.20192	0.13245	5
[3]	108 Coleridge Rd	52.19093	0.14592	4
[4]	114 Vinery Rd	52.19999	0.15257	9
[6]	OP 6 Station Rd	52.194855	0.132876	3
[7]	151/153 Coldhams Ln	52.20377	0.15201	10
[10]	O/S ARU East Road	52.20407	0.13294	6
[12]	55 Devonshire Rd	52.19624	0.13713	2
[13]	214 Milton Rd	52.22063	0.13416	14
[14]	140 Hills Rd	52.19008	0.13534	7
[15]	560 Newmarket Road	52.21288	0.15674	13
[16]	142 Perne Road	52.19239	0.15467	12
[40]	117 Cherry Hinton Rd	52.18832	0.14227	8
[41]	2 Tenison Rd	52.19994	0.13679	1
	Mill Road Bridge	52.198675	0.14113	

**Table 2:** Case processing summary of ANN for three scenarios of the Mill Road Bridge closure.

	Before		During		After	
Partitions	Number	Percent	Number	Percent	Number	Percent
Training	8992	47.9%	19,998	48.0%	150,200	47.6%
Testing	6032	32.1%	11,986	28.8%	90,092	28.5%
Validation	3764	20.0%	9681	23.2%	75,576	23.9%
Total	18,788	100.0%	41,665	100.0%	315,868	100.0%

**Table 3:** Performance summary of ANN models for three scenarios.

Partitions	Error Parameters	Before	During	After
Training	SSE	7.140	12.981	39.157
	RE	0.208	0.210	0.221
Testing	SSE	5.484	7.666	23.167
	RE	0.244	0.221	0.225
Validation	RE	0.217	0.238	0.220

**Table 4:** Factors affecting the spatial distribution of bicycles before the closure of the Mill Road Sensor Project.

Variables	Importance	Normalised Importance	Coefficient Standard Errors	Sig. Level	95% Confidence Level	
					Lower Bound	Upper Bound
Ranking of sensors distance proximity to road closure	0.130	39.2%	0.074	0.000	−0.602	−0.310
Presence of rush hour traffic	0.066	19.9%	0.377	0.000	10.647	12.126
Days of the week	0.016	4.8%	0.594	0.000	6.654	8.982
Number of cars per hour	0.072	21.6%	0.003	0.001	0.003	0.014
Number of pedestrians per hour	0.333	100.0%	0.007	0.000	0.209	0.235
Number of motor bikes per hour	0.146	43.9%	0.049	0.000	1.807	1.998
Number of buses per hour	0.113	33.8%	0.042	0.000	1.565	1.729
Number of large rigid vehicles (2 and 3 axles) per hour	0.047	14.1%	0.096	0.000	0.363	0.738
Number of large rigid vehicles (4 axles and more) per hour	0.061	18.3%	0.218	0.000	−1.704	−0.850
Number of light commercial vehicles per hour	0.017	5.0%	0.018	0.321	−0.054	0.018

**Table 5:** Factors affecting the spatial distribution of bicycles during the closure of the Mill Road Sensor Project.

Variables	Importance	Normalised Importance	Coefficient Standard Errors	Sig. Level	95% Confidence Level	
					Lower Bound	Upper Bound
Ranking of sensors distance proximity to road closure	0.252	99.1%	0.055	0.000	−1.306	−1.090
Presence of rush hour traffic	0.071	28.0%	0.279	0.000	17.490	18.582
Days of the week	0.026	10.1%	0.447	0.000	6.641	8.393
Number of cars per hour	0.068	26.6%	0.002	0.000	−0.044	−0.036
Number of pedestrians per hour	0.254	100.0%	0.004	0.000	0.153	0.169
Number of motor bikes per hour	0.072	28.4%	0.040	0.000	2.814	2.972
Number of buses per hour	0.037	14.6%	0.034	0.000	0.659	0.793
Number of large rigid vehicles (2 & and axles) per hour	0.129	51.0%	0.070	0.001	0.102	0.376
Number of large rigid vehicles (4 axles and more) per hour	0.047	18.5%	0.213	0.000	1.326	2.162
Number of light commercial vehicles per hour	0.045	17.7%	0.012	0.000	0.084	0.131

**Table 6:** Factors affecting the spatial distribution of bicycles after the opening of the Mill Road Sensor Project.

Variables	Importance	Normalised Importance	Coefficient Standard Errors	Sig. Level	95% Confidence Level	
					Lower Bound	Upper Bound
Ranking of sensors distance proximity to road closure	0.122	28.1%	0.016	0.000	−0.539	−0.478
Presence of rush hour traffic	0.037	8.5%	0.080	0.000	5.912	6.224
Days of the week	0.009	2.0%	0.125	0.000	2.637	3.128
Number of cars per hour	0.060	13.8%	0.001	0.067	−0.002	0.000
Number of pedestrians per hour	0.435	100.0%	0.001	0.000	0.265	0.270
Number of motor bikes per hour	0.073	16.8%	0.009	0.000	1.256	1.293
Number of buses per hour	0.063	14.5%	0.012	0.000	0.858	0.904
Number of large rigid vehicles (2 and 3 axles) per hour	0.106	24.4%	0.022	0.000	−0.424	−0.339
Number of large rigid vehicles (4 axles and more) per hour	0.043	9.9%	0.064	0.000	0.358	0.608
Number of light commercial vehicles per hour	0.052	12.0%	0.004	0.000	0.200	0.214

## Data Availability

The Mill Road closure traffic sensor data can be publicly viewed on the Cambridgeshire Insight open data portal.

## Abbreviations

The following abbreviations are used in this manuscript:

ANN	Multidisciplinary Digital Publishing Institute
RC	Recurrent Congestion
NRC	Non-Recurrent Congestion
LD	Linear dichroism
HGVs	Heavy goods vehicles
GPS	Global Positioning System
GDR	Generalised Delta Rule
MEMS	Micro-Electro-Mechanical Systems
WSNs	wireless sensor networks
CCTs	Constructing causality trees
CBD	Central business district
STARIMA	Space–Time Autoregressive Integrated Moving Average
LTNs	Low-traffic neighbourhoods

## Appendix A

**Table A1:** Parameter estimates of input variables before the closure of the Mill Road Bridge.

Predictor		Predicted																
		Hidden Layer 1									Hidden Layer 2							Output Layer
		H(1:1)	H(1:2)	H(1:3)	H(1:4)	H(1:5)	H(1:6)	H(1:7)	H(1:8)	H(1:9)	H(2:1)	H(2:2)	H(2:3)	H(2:4)	H(2:5)	H(2:6)	H(2:7)	Cyclist
Input Layer	(Bias)	−0.871	0.835	−0.124	−0.350	0.109	−0.136	−0.214	−0.095	1.051								
	Sensor41	0.270	−0.218	−0.080	−0.148	0.342	0.465	−0.450	−0.292	0.615								
	Sensor12	0.775	−0.792	−0.148	−0.057	−0.157	0.113	0.072	0.500	−0.889								
	Sensor6	−0.732	0.725	−0.541	0.222	−0.244	0.021	−0.330	−0.102	0.718								
	Sensor3	0.263	−0.207	0.084	−0.405	0.398	0.024	−0.475	−0.026	0.288								
	Sensor2	−0.221	0.112	−0.582	0.225	0.429	0.478	0.366	−0.466	0.850								
	Sensor10	0.076	0.789	0.209	0.121	0.138	0.275	0.509	−0.619	0.855								
	Sensor14	0.096	−0.522	0.063	0.233	0.286	−0.128	−0.560	0.275	−0.329								
	Sensor40	0.250	0.453	−0.197	0.046	0.264	−0.203	−0.353	−0.325	−0.243								
	Sensor4	−0.228	0.289	−0.155	0.327	−0.017	0.145	−0.328	0.105	−0.176								
	Sensor7	−0.053	−0.063	−0.099	0.154	−0.124	−0.279	−0.347	0.248	0.119								
	Sensor1	0.771	−0.275	0.276	−0.542	0.004	0.218	−0.261	−0.093	−0.153								
	Sensor16	−0.149	0.393	−0.449	−0.290	0.058	0.206	0.347	0.214	0.331								
	Sensor15	−0.707	−0.183	−0.149	0.541	0.302	0.530	−0.462	−0.084	0.036								
	Sensor13	0.034	0.275	−0.056	−0.263	0.081	0.409	−0.127	0.091	0.488								
	Rush Hrs	0.938	−0.351	−0.480	0.170	−0.037	−0.483	0.100	−0.120	−0.122								
	weekdays	−0.556	−0.119	0.008	0.048	0.251	−0.245	−0.109	0.478	−0.209								
	Cars	0.609	0.331	0.354	−0.429	0.242	0.439	−0.367	0.320	0.242								
	Motorbikes	0.345	0.202	0.566	0.061	0.464	0.566	0.345	−0.341	−0.676								
	Buses	−0.292	−0.259	0.430	−0.400	−0.063	−0.505	−0.264	0.209	0.134								
	OGV1	−0.168	−0.253	0.011	0.278	−0.317	−0.301	−0.359	0.404	0.243								
	OGV2	−0.203	−0.518	0.037	0.370	0.216	0.199	0.005	−0.770	−0.513								
	LGV	0.057	−0.041	0.215	−0.030	−0.132	0.026	−0.050	0.080	0.314								
	Pedestrians	0.466	−0.102	−0.614	−0.331	0.308	−0.548	0.343	0.931	0.049								
Hidden Layer 1	(Bias)										−0.145	0.628	0.173	0.274	−0.261	0.279	0.675	
	H(1:1)										−0.674	−0.394	−0.552	−1.079	−0.981	−1.590	−1.529	
	H(1:2)										−0.117	0.158	0.836	1.427	0.776	0.588	0.583	
	H(1:3)										0.355	0.243	−0.867	−0.726	−0.609	−0.444	−0.092	
	H(1:4)										0.315	−0.118	−0.120	0.421	0.161	0.568	0.433	
	H(1:5)										0.302	−0.111	0.102	−0.176	−0.469	−0.107	−0.638	
	H(1:6)										−0.033	−0.043	0.245	0.900	0.060	0.539	0.819	
	H(1:7)										0.210	−0.242	0.234	0.743	−0.032	0.009	0.119	
	H(1:8)										−0.239	−0.193	−0.774	−0.836	−0.207	−0.684	−1.199	
	H(1:9)										0.382	0.858	1.022	1.679	0.595	0.837	0.907	
Hidden Layer 2	(Bias)																	2.306
	H(2:1)																	0.274
	H(2:2)																	0.435
	H(2:3)																	−0.836
	H(2:4)																	−2.633
	H(2:5)																	−1.172
	H(2:6)																	−1.586
	H(2:7)																	−2.081

**Table A2:** Parameter estimates of input variables during the closure of the Mill Road Bridge.

Predictor		Predicted																
		Hidden Layer 1									Hidden Layer 2							Output Layer
		H(1:1)	H(1:2)	H(1:3)	H(1:4)	H(1:5)	H(1:6)	H(1:7)	H(1:8)	H(1:9)	H(2:1)	H(2:2)	H(2:3)	H(2:4)	H(2:5)	H(2:6)	H(2:7)	Cyclist
Input Layer	(Bias)	−0.691	−0.152	−0.121	0.330	−0.317	0.462	0.226	0.453	−0.486								
	Sensor41	−0.405	0.240	−0.405	0.266	0.028	−0.040	0.600	0.258	−0.119								
	Sensor12	1.618	0.736	0.377	−0.944	1.049	−0.519	−0.417	−0.791	−0.229								
	Sensor6	−0.384	−0.528	−0.986	0.135	−0.758	0.026	0.670	0.319	−0.266								
	Sensor3	0.016	−0.276	0.210	0.066	0.389	−0.427	0.115	−0.022	0.123								
	Sensor2	−0.347	−0.416	−0.377	0.491	0.012	0.237	−0.120	−0.084	0.256								
	Sensor10	−1.057	−0.068	−0.159	0.079	−0.414	0.336	0.028	0.743	0.512								
	Sensor14	0.917	0.686	0.187	−0.211	0.530	−0.386	0.126	0.074	0.221								
	Sensor40	0.124	0.508	0.415	0.076	−0.412	−0.339	−0.403	0.057	0.113								
	Sensor4	0.018	−0.045	−0.457	0.308	0.074	0.510	0.421	−0.146	−0.387								
	Sensor7	0.210	−0.484	−0.451	−0.160	−0.461	0.479	0.194	0.138	0.216								
	Sensor1	0.213	0.533	0.141	−0.219	0.165	−0.149	0.030	0.246	0.063								
	Sensor16	−0.270	−0.009	−0.505	0.323	−0.310	−0.003	0.253	0.375	−0.398								
	Sensor15	−0.602	0.053	−0.580	0.375	0.117	0.158	0.489	0.600	−0.342								
	Sensor13	−0.355	0.433	0.223	0.206	−0.514	−0.326	0.037	−0.034	−0.343								
	Rush Hrs	0.060	0.666	0.774	0.286	−0.141	−0.442	−0.228	−0.716	−0.479								
	weekdays	0.423	0.186	0.600	0.093	0.425	−0.107	0.167	0.468	0.574								
	Cars	−0.050	−0.063	−0.163	−0.384	−0.341	−0.514	−0.271	−0.626	0.111								
	Pedestrians	−0.172	−0.012	−0.791	−1.502	−0.347	−0.864	−1.065	−0.591	−0.849								
	Motorbikes	0.375	1.196	−0.132	−0.054	0.323	0.534	0.273	−0.002	0.185								
	Buses	−0.304	−0.280	0.316	0.220	−0.162	−0.486	−0.224	−0.033	−0.931								
	OGV1	0.483	−0.444	0.279	−0.433	0.159	0.718	0.479	0.064	−0.187								
	OGV2	0.331	−0.113	−0.075	0.046	0.339	0.411	−0.237	0.315	0.415								
	LGV	−0.244	0.480	0.038	0.537	0.027	−0.337	0.012	0.149	−0.331								
Hidden Layer 1	(Bias)										0.766	−0.533	0.911	−0.047	−0.387	0.221	−0.274	
	H(1:1)										−0.870	−0.596	−1.834	−1.187	0.136	0.785	−0.325	
	H(1:2)										−0.782	−0.832	−0.146	−0.134	−0.025	0.548	−0.003	
	H(1:3)										−0.577	−0.669	−0.419	−0.651	−0.279	0.152	−0.876	
	H(1:4)										0.624	0.058	1.497	0.586	−0.208	−0.303	0.517	
	H(1:5)										−0.815	−0.051	−0.838	−0.216	0.198	0.314	−0.596	
	H(1:6)										0.472	0.687	1.088	0.217	−0.506	−0.505	0.141	
	H(1:7)										0.803	0.397	1.040	0.368	−0.090	−0.166	−0.427	
	H(1:8)										0.395	0.565	1.291	−0.233	0.146	−0.594	−0.057	
	H(1:9)										0.650	0.253	0.555	0.535	−0.437	−0.176	−0.161	
Hidden Layer 2	(Bias)																	1.122
	H(2:1)																	−1.936
	H(2:2)																	−1.075
	H(2:3)																	−3.146
	H(2:4)																	−1.154
	H(2:5)																	0.533
	H(2:6)																	1.667
	H(2:7)																	−0.524

**Table A3:** Parameter estimates of input variables after the closure of the Mill Road Bridge.

Predictor		Predicted																
		Hidden Layer 1									Hidden Layer 2							Output Layer
		H(1:1)	H(1:2)	H(1:3)	H(1:4)	H(1:5)	H(1:6)	H(1:7)	H(1:8)	H(1:9)	H(2:1)	H(2:2)	H(2:3)	H(2:4)	H(2:5)	H(2:6)	H(2:7)	Cyclist
Input Layer	(Bias)	0.617	0.071	−0.457	0.161	−0.732	0.705	−0.223	0.428	0.491								
	Sensor41	0.320	0.472	−0.644	0.205	0.137	0.290	0.127	0.348	−0.018								
	Sensor12	−0.805	−0.109	0.279	0.071	0.502	−0.790	0.385	0.092	−0.356								
	Sensor6	1.222	0.136	−0.303	−0.899	−0.376	0.744	0.172	−0.170	0.079								
	Sensor3	−0.690	0.446	−0.062	0.278	0.106	−0.135	−0.267	−0.415	0.400								
	Sensor2	0.226	0.428	0.405	0.152	−0.634	−0.086	−0.123	−0.004	0.386								
	Sensor10	0.807	0.359	−0.754	0.181	−0.029	0.566	0.225	0.158	−0.164								
	Sensor14	−0.529	−0.533	0.502	−0.037	0.524	−0.376	−0.228	0.352	−0.553								
	Sensor40	−0.126	−0.273	−0.030	0.350	−0.285	−0.523	−0.380	−0.147	0.292								
	Sensor4	−0.197	0.030	−0.162	−0.203	−0.203	0.274	−0.397	−0.354	0.369								
	Sensor7	0.406	−0.216	−0.342	0.026	0.197	0.282	0.108	−0.083	−0.412								
	Sensor1	−0.195	0.513	0.099	0.003	−0.255	−0.286	0.032	0.555	−0.354								
	Sensor16	0.535	−0.303	0.053	−0.023	−0.117	0.419	−0.114	0.161	−0.028								
	Sensor15	0.303	−0.010	−0.472	−0.283	−0.109	0.593	−0.356	0.432	−0.243								
	Sensor13	−0.148	0.185	−0.807	−0.761	−0.029	0.068	−0.043	−0.557	−0.264								
	Rush Hrs	−0.220	0.207	−0.420	0.531	0.348	−0.515	−0.152	−0.111	0.085								
	weekdays	−0.243	−0.312	0.022	0.533	0.224	0.859	0.366	−0.236	−0.249								
	Cars	0.229	−0.425	−0.823	0.774	0.534	−0.123	0.027	0.269	−0.481								
	Pedestrians	−0.113	0.013	0.857	−0.145	−0.492	−0.459	1.892	0.859	0.444								
	Motorbikes	0.022	−0.352	−0.103	−0.329	0.428	−0.099	0.124	0.377	−0.044								
	Buses	−0.138	0.252	0.365	−0.045	0.173	0.148	−0.262	0.017	−0.142								
	OGV1	−0.359	0.068	0.377	−0.301	−0.187	−0.164	−0.496	−0.100	0.203								
	OGV2	−0.086	−0.009	−0.046	−0.055	−0.141	−0.017	0.139	−0.397	−0.125								
	LGV	−0.021	0.148	0.149	−0.147	0.271	0.545	0.417	−0.162	−0.561								
Hidden Layer 1	(Bias)										−0.284	−0.202	−0.328	−0.002	0.579	0.192	−0.444	
	H(1:1)										0.106	0.424	0.938	0.538	1.298	1.050	−0.948	
	H(1:2)										−0.181	−0.245	0.425	0.263	0.132	0.242	−0.087	
	H(1:3)										−0.163	−0.616	−0.282	−0.320	−1.312	−0.519	0.651	
	H(1:4)										−0.327	−0.145	−0.065	−0.638	−0.532	−0.663	0.708	
	H(1:5)										0.351	−0.639	−0.704	−0.559	0.081	−0.837	0.818	
	H(1:6)										−0.043	0.777	0.632	1.055	1.781	1.192	−0.354	
	H(1:7)										0.055	−0.543	−0.926	−0.445	−0.731	−0.061	0.473	
	H(1:8)										−0.289	−0.151	0.097	−0.514	0.010	−0.136	0.032	
	H(1:9)										−0.373	0.136	0.579	0.253	0.340	−0.024	−0.599	
Hidden Layer 2	(Bias)																	0.276
	H(2:1)																	0.287
	H(2:2)																	−1.057
	H(2:3)																	−1.284
	H(2:4)																	−1.387
	H(2:5)																	−2.585
	H(2:6)																	−1.564
	H(2:7)																	2.474
